# MHY2245, a Sirtuin Inhibitor, Induces Cell Cycle Arrest and Apoptosis in HCT116 Human Colorectal Cancer Cells

**DOI:** 10.3390/ijms23031590

**Published:** 2022-01-29

**Authors:** Yong Jung Kang, Jung Yoon Jang, Young Hoon Kwon, Jun Ho Lee, Sanggwon Lee, Yujin Park, Young-Suk Jung, Eunok Im, Hyung Ryong Moon, Hae Young Chung, Nam Deuk Kim

**Affiliations:** 1 Department of Pharmacy, Research Institute for Drug Development, College of Pharmacy, Pusan National University, Busan 46241, Korea; biogreen80@hotmail.com (Y.J.K.); jungyoon486@pusan.ac.kr (J.Y.J.); k_8010@naver.com (Y.H.K.); justin4117@pusan.ac.kr (J.H.L.); youngjung@pusan.ac.kr (Y.-S.J.); eoim@pusan.ac.kr (E.I.); hyjung@pusan.ac.kr (H.Y.C.); 2 Department of Manufacturing Pharmacy, Research Institute for Drug Development, College of Pharmacy, Pusan National University, Busan 46241, Korea; lsk3232@pusan.ac.kr (S.L.); pyj10@pusan.ac.kr (Y.P.); mhr108@pusan.ac.kr (H.R.M.)

**Keywords:** SIRT inhibitor, sirtinol, DNA damage response, cell cycle arrest, apoptosis, colorectal cancer cells

## Abstract

Sirtuins (SIRTs), which are nicotinamide adenine dinucleotide-dependent class III histone deacetylases, regulate cell division, survival, and senescence. Although sirtinol, a synthetic SIRT inhibitor, is known to exhibit antitumor effects, its mechanism of action is not well understood. Therefore, we aimed to assess the anticancer effects and underlying mechanism of MHY2245, a derivative of sirtinol, in HCT116 human colorectal cancer cells in vitro. Treatment with MHY2245 decreased SIRT1 activity and caused DNA damage, leading to the upregulation of p53 acetylation, and increased levels of p53, phosphorylation of H2A histone family member X, ataxia telangiectasia and Rad3-related kinase, checkpoint kinase 1 (Chk1), and Chk2. The level of the breast cancer type 1 susceptibility protein was also found to decrease. MHY2245 induced G2/M phase cell cycle arrest via the downregulation of cyclin B1, cell division cycle protein 2 (Cdc2), and Cdc25c. Further, MHY2245 induced HCT116 cell death via apoptosis, which was accompanied by internucleosomal DNA fragmentation, decreased B-cell lymphoma 2 (Bcl-2) levels, increased Bcl-2-asscociated X protein levels, cleavage of poly(ADP-ribose) polymerase, and activation of caspases -3, -8, and -9. Overall, MHY2245 induces cell cycle arrest, triggers apoptosis through caspase activation, and exhibits DNA damage response-associated anticancer effects.

## 1. Introduction

Colorectal cancer (CRC), also known as bowel and colon cancer, is the third leading cause of cancer deaths in both men and women, and has recently become the third most common cancer in the United States [1]. According to the 2020 cancer incidence and mortality predictions in Korea, CRC is the third most common cancer for both men and women, the expected mortality rate for men is the third, and the mortality rate for women is the second [2]. Currently, effective treatment methods for CRC include surgery, chemotherapy, radiation, or a combination of chemotherapy and radiotherapy. Although more than 50% of CRC patients who underwent surgical resection are cured, 40–50% of these patients finally recur, and the chances of the patient returning to work are very low [3]. Early diagnosis and treatment of this disease increases the patient’s chances of survival, emphasizing the importance of appropriate and accurate treatment [4]. A continuing need thus exists for assessing and developing potential prophylactic agents for CRC.

Sirtuins (SIRT 1–7) are a family of nicotinamide adenine dinucleotide (NAD^+^)-dependent protein-modifying enzymes with activity in lysine deacetylation, adenosine diphosphate (ADP)-ribosylation, and/or deacylation. These enzymes are involved in important cellular processes, including cell survival, stress response, metabolism, senescence, aging, and tumorigenesis, through the deacetylation of several substrates [5,6]. SIRT1 is the most extensively researched enzyme of the SIRT family and is largely related to the adjustment of the cellular process that determines life span, including anti-apoptosis, neuronal protection, cellular senescence, and aging. Importantly, SIRT1 is upregulated in several cancers, including leukemia, lymphoma, soft-tissue sarcomas, prostate cancer, lung and colon carcinomas, non-melanoma skin cancers, and more [5]. The overexpression of SIRT1 in cancer is associated with the need for chemotherapy, development of resistance to ionizing radiation, and silencing of tumor suppressor genes [7]. Recent studies have shown that p53 is not only the target of SIRT1, but also of SIRT2. SIRT2 deacetylates histone H3 lysine 56, and α-tubulin shares the non-histone substrates, forkhead box proteins O1 and O3, and p53 with SIRT1, thereby controlling the cancer cell cycle [8]. SIRT1 is related to DNA damage repair and cell cycle arrest after DNA damage [9,10]. SIRT1 attenuates p53-dependent apoptosis in CRC cells [11], deactivates p53 through deacetylation of the lysine 382 residue of p53, and negatively regulates its transactivation activity [12]. However, the role of SIRT1 in CRC has not yet been fully elucidated [13,14,15].

**Figure 1:**
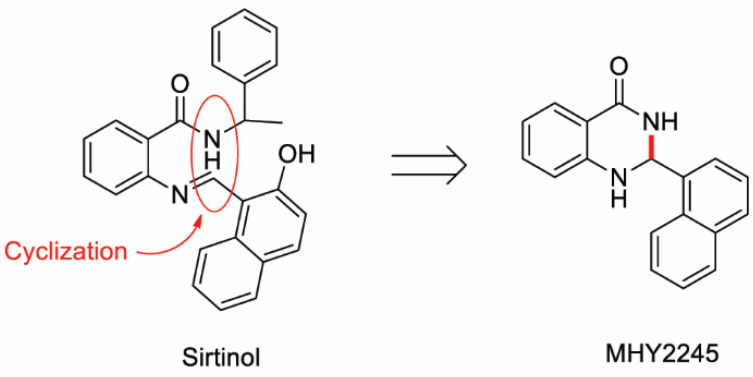
Synthetic scheme for MHY2245. Structures of sirtinol and MHY2245 [2-(naphthalen-1-yl)-2,3-dihydroquinazolin-4(1H)-one].

Many small-molecule inhibitors of SIRTs, such as splitomicin [16], dihydrocoumarin [17], indoles [18], cambinol [19], and sirtinol [20], have been discovered. Sirtinol is a class III histone deacetylase (HDAC) inhibitor that does not inhibit class I or II HDAC [16,21]. Sirtinol is a dual SIRT1/SIRT2 inhibitor and a cell-permeable six-membered lactone ring derived from naphthol [22]. Sirtinol induces growth arrest in human non-small cell lung carcinoma cells, breast cancer cells, leukemia cells, and CRC cells [8,23,24,25,26]. Sirtinol also induces apoptosis in lung cancer cells [27], adult T-cell leukemia cells [26], and breast cancer cells [25]. Sirtinol is a well-known SIRT inhibitor; however, it was found to have moderate IC_50_ values of 48–131 μM [28]. Structural modification of sirtinol by bundling two branches in the phenyl ring has been attempted to improve its efficacy. Therefore, we studied the synthesized sirtinol derivative, MHY2245 [2-(naphthalen-1-yl)-2,3-dihydroquinazolin-4(1H)-one] (Figure 1) [29], and investigated its effects on cell cycle regulation, induction of apoptosis, and DNA damage response in HCT116 [wild-type (WT) *TP53*], HT-29 (mutant *TP53*), DLD-1 (mutant *TP53*) human CRC cells, and NCM460D (an epithelial cell line from a normal human colon).

## 2. Results

### 2.1. Effects of MHY2245 on the Growth of HCT116 Cells

**Figure 2:**
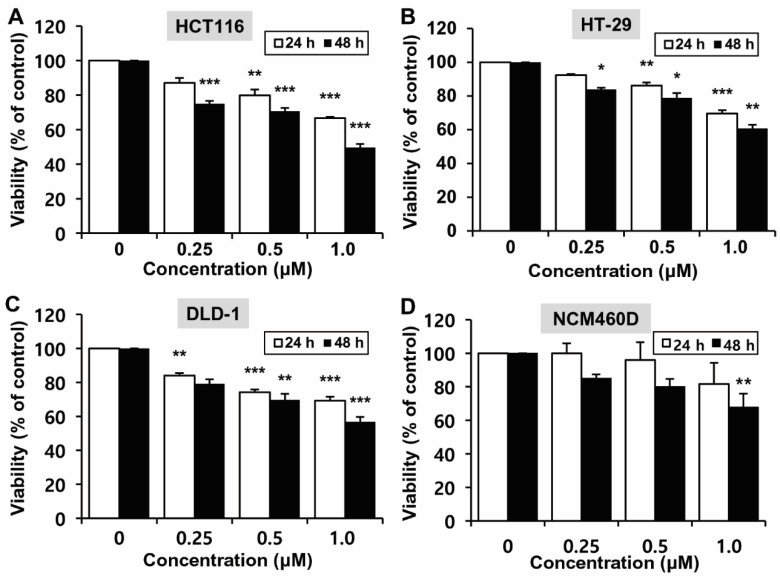
Effect of MHY2245 on the viability of the human colorectal cancer cell lines HCT116, HT-29, and DLD-1, and the normal human colon epithelial cell line, NCM460D. Graphs showing the viability of HCT116 (**A**), HT-29 (**B**), DLD-1 (**C**), and NCM460D (**D**) cells treated with increasing concentrations of MHY2245 for 24 h and 48 h. Results are presented as mean ± standard deviation (SD), *n* = 3, and are expressed as a percentage of viability in vehicle-treated control cells. * *p* < 0.05, ** *p* < 0.01, and *** *p* < 0.001 compared with vehicle-treated control cells.

To determine the effects of MHY2245 on the growth of CRC cells (HCT116, HT-29 and DLD-1) with different p53 types, the 3-(4,5-dimethylthiazol-2-yl)-2,5-diphenyl tetrazolium bromide (MTT) assay was performed. Although treatment with MHY2245 decreased cell growth in time- and concentration-dependent manners in the three different cell lines, HCT116 cells with WT *TP53* were more sensitive to MHY2245 than HT-29 and DLD-1 cells with mutant *TP53* (Figure 2A–C). Further, MHY2245 was less cytotoxic to cancer cells in the epithelial cell line, NCM460D, derived from a normal human colon (Figure 2D). Based on these findings, HCT116 cells were used in further experiments.

### 2.2. Molecular Docking Simulation of SIRT1/2 with MHY2245

**Figure 3:**
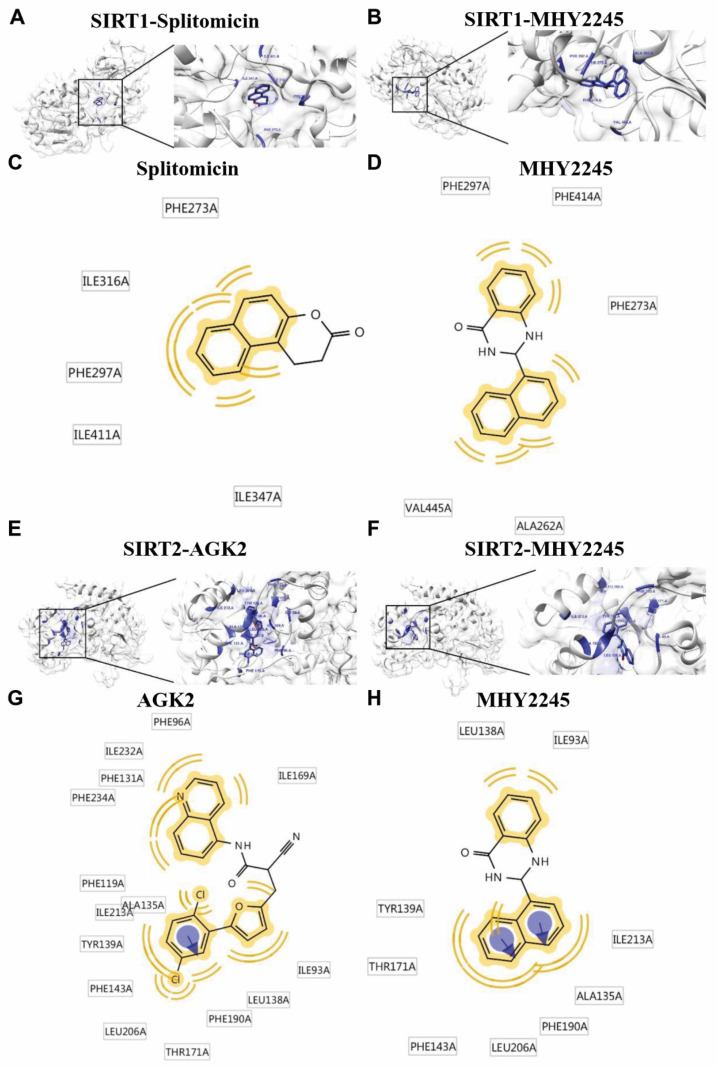
Docking simulation of MHY2245, splitomicin, AGK2 with SIRT1/2, and pharmacophore analysis. Docking simulation results between splitomicin (**A**) and MHY2245 (**B**) toward human SIRT1. Pharmacophore results for splitomicin (**C**) and MHY2245 (**D**) obtained using LigandScout 4.1.5 showing hydrophobic (yellow) interactions. Docking simulation results between AGK2 (**E**) and MHY2245 (**F**) toward human SIRT2. Pharmacophore results for AGK2 (**G**) and MHY2245 (**H**) obtained using LigandScout 4.1.5 showing hydrophobic (yellow) interactions and aromatic interactions (violet arrow).

To further assess the effect of MHY2245 on SIRT1/2, a protein-ligand docking simulation was performed using the AutoDock Vina, AutoDock 4, and Dock 6 programs. The predicted 3D structures of SIRT1, splitomicin (SIRT1 inhibitor) (Figure 3A), and MHY2245 (Figure 3B) are shown, with black lines indicating splitomicin and MHY2245, respectively (Figure 3A,B). The docking simulation was effective and led to a considerable score. The binding energies of splitomicin and SIRT1 were −9.6 (AutoDock Vina), −7.63 (AutoDock 4), and −28.46 (Dock 6) kcal/mol, while those of MHY2245 and SIRT 1 were −9.7 (AutoDock Vina), −9.7 (AutoDock 4), and −33.47 (Dock 6) kcal/mol, respectively (Table 1). Splitomicin interacted with SIRT 1 via 5 Van der Waals bond interactions, and MHY2245 interacted via 5 Van der Waals bond interactions (Figure 3C,D). These binding energy data indicate that MHY2245 exhibited a greater binding affinity than splitomicin with SIRT1.

The predicted 3D structures of SIRT2, AGK2 (SIRT2 inhibitor) (Figure 3E), and MHY2245 (Figure 3F) are shown, with black lines indicating AGK2 and MHY2245, respectively (Figure 3E,F). The docking simulation was effective and had a considerable score. The binding energies of AGK2 and SIRT2 were −10.7 (AutoDock Vina), −12.7 (AutoDock 4), and −36.66 (Dock 6) kcal/mol, while those of MHY2245 and SIRT2 were −10.9 (AutoDock Vina), −9.99 (AutoDock 4), and −32.00 (Dock 6) kcal/mol, respectively (Table 2). AGK2 interacted with SIRT2 via 15 Van der Waals bond interactions and one aromatic interaction, while MHY2245 interacted with SIRT2 via nine Van der Waals bond interactions and two aromatic interactions (Figure 3G,H). These binding energy data indicate that MHY2245 exhibited a lower binding affinity than AGK2 with SIRT2. Overall, MHY2245 is predicted to bind more strongly to SIRT1 than SIRT2 and inhibit its activity.

### 2.3. SIRT1/2 Inhibitory Effect and Induction of DNA Damage of MHY2245 in HCT116 Cells

**Figure 4:**
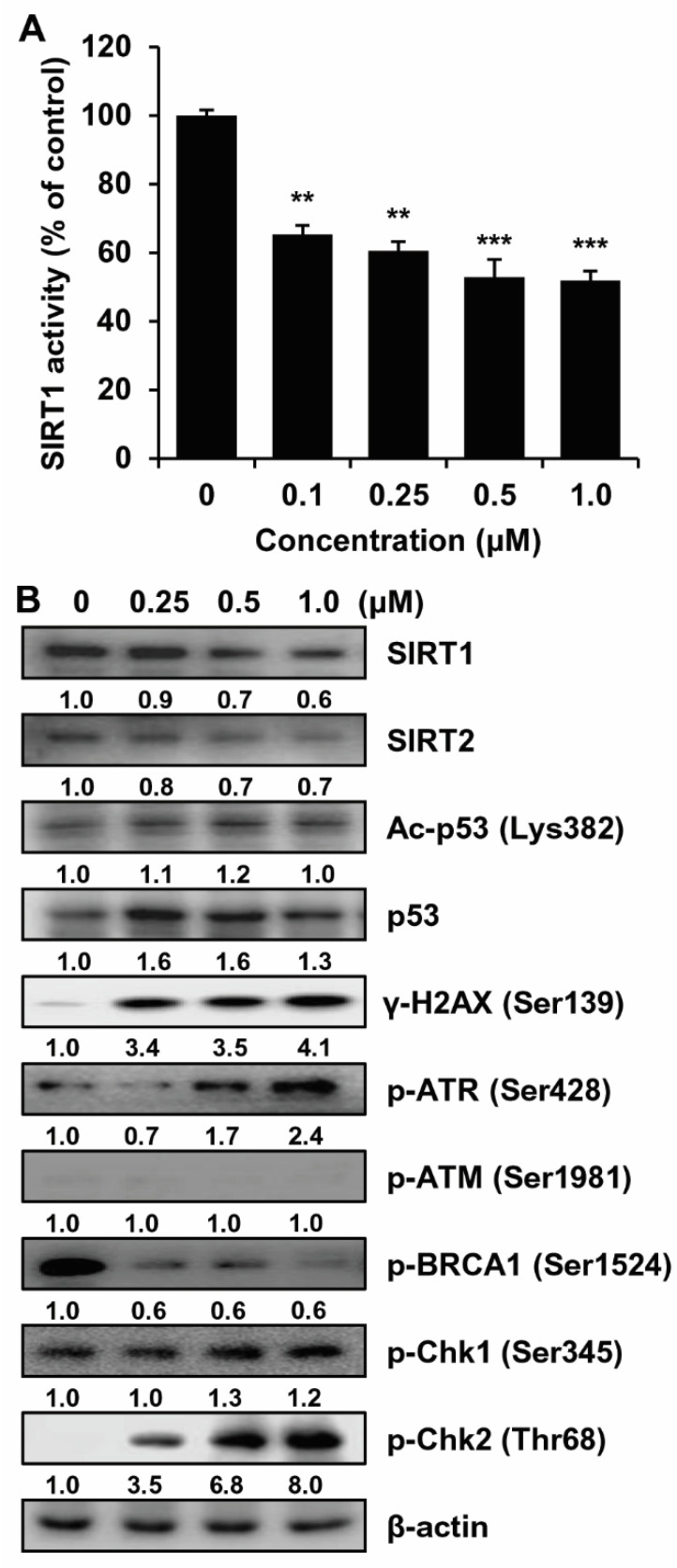
Effect of MHY2245 on SIRT and the expression of DNA damage response mediators and effector cascade in HCT116 cells. (**A**) Graph showing SIRT1 activity. Results are presented as mean ± SD, *n* = 3, and are expressed as a percentage of SIRT1 activity in vehicle-treated control cells. ** *p* < 0.01 and *** *p* < 0.001 compared with vehicle-treated control cells. (**B**) Cells were treated with the indicated concentrations of MHY2245 for 24 h and Western blot analyses were conducted to investigate the expression of SIRT1, SIRT2, Ac-p53 (Lys382), p53, γ-H2AX (Ser139), p-ATR (Ser428), p-ATM (Ser1981), p-BRCA1 (Ser1524), p-Chk1 (Ser345), and p-Chk2 (Thr68). β-actin was used as a loading control. Representative results from three independent experiments are shown.

Several studies have revealed that SIRT1 inhibitors inhibit tumor formation [30]. The activity of the sirtinol derivative, MHY2245, a suspected inhibitor of SIRT1, was measured to confirm its inhibitory activity on SIRT. As shown in Figure 4A, MHY2245 remarkably inhibited SIRT1 enzyme activity in a concentration-dependent manner. The effects of MHY2245 on SIRT protein levels were subsequently investigated via Western blot analysis. MHY2245 decreased the expression levels of SIRT1 and SIRT2 in a concentration-dependent manner. MHY2245 increased the levels of acetylated Lys382 in p53 (Ac-p53, Lys382) and p53 (Figure 4B). A previous study reported increased DNA damage when cancer cells were treated with HDAC inhibitors alone or in combination with other drugs [31]. Another study showed that sirtuin regulates the biological processes of DNA damage and repair [32]. Based on previous research, we investigated the relationship between SIRT inhibition and DNA damage response. Western blot analyses revealed that treatment with MHY2245 resulted in increased levels of gamma-H2A histone family member X (γ-H2AX)—a marker of double-strand breaks (Figure 4B). We further investigated the association between these findings and the DNA damage response signaling pathway.

DNA damage response is initiated by a variety of protein kinases and other factors, such as ataxia telangiectasia and Rad3-related kinase (ATR), and ataxia telangiectasia mutated kinase (ATM) [33]. These kinases and factors recognize DNA damage and phosphorylate downstream molecules, such as checkpoint kinase 1 (Chk1), Chk2, p53, and associated factors involved in the process of cell cycle, DNA repair, and apoptosis [34]. As shown in Figure 4B, MHY2245 treatment in HCT116 cells markedly induced the phosphorylation of ATR (Ser428), Chk1 (Ser345), and Chk2 (Thr68) in a concentration-dependent manner, although p-breast cancer type 1 susceptibility protein (BRCA1, Ser1524), which is a DNA repair-associated protein, was significantly downregulated. Moreover, there was no change in the expression of p-ATM (Ser1981). These experiments were repeated several times to confirm the lack of change in the expression of p-ATM (Ser1981). However, the expression of p-ATM (Ser1981) was upregulated when cells were treated with a topoisomerase inhibitor (data not shown). Although there was no increase in the expression of p-ATM (Ser1981), increased phosphorylation of ATM (Ser1981) target molecules, such as H2AX (Ser139), Chk1 (Ser345), Chk2 (Thr68) and p53, and decreased phosphorylation of BRCA1 (Ser1524) suggested that there were some changes that were not observed herein. In summary, these results indicate that MHY2245 is a SIRT inhibitor and SIRT inhibition leads to enhanced DNA damage in response to MHY2245 treatment in HCT116 cells.

### 2.4. Effects of MHY2245 on the Cell Cycle in HCT116 Cells

**Figure 5:**
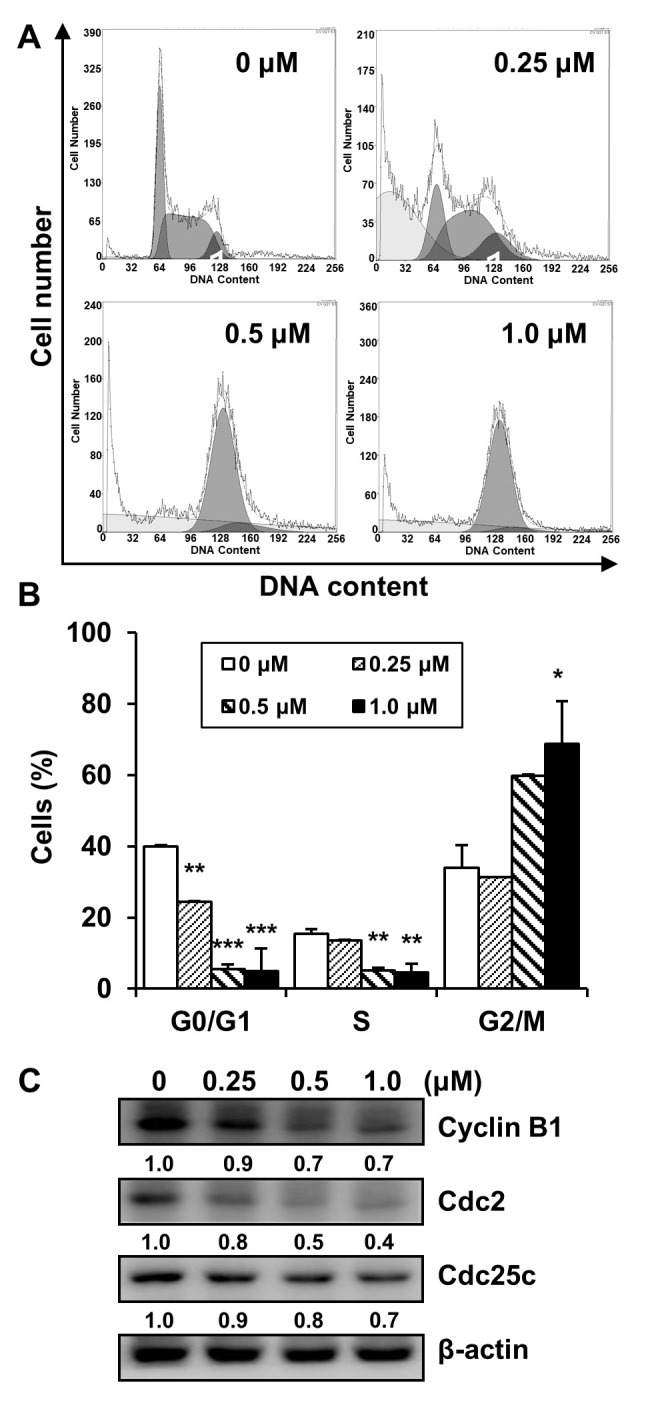
Effect of MHY2245 on cell cycle modulation in HCT116 cells. (**A**) Exponentially growing cells at 60–70% confluence were treated with the indicated concentrations of MHY2245 for 24 h. Propidium iodide (PI)-stained cells were subjected to flow cytometry analysis to determine the cell distribution at each stage of the cell cycle. (**B**) The percentage of cells in the G0/G1, S, and G2/M phases of the cell cycle was calculated and is displayed on each bar graph. Results are presented as mean ± SD, *n* = 3, and are expressed as a percentage of that in vehicle-treated control cells. * *p* < 0.05, ** *p* < 0.01, and *** *p* < 0.001 compared with vehicle-treated control cells. (**C**) Effects of MHY2245 on the expression of cell cycle regulatory proteins analyzed via Western blot analysis of total lysates of cells treated with increasing concentrations of MHY2245. The membranes were probed with Cyclin B1, Cdc2, and Cdc25c. β-actin was used as a loading control. Representative results from three independent experiments are shown.

To determine whether MHY2245 affects cell cycle distribution, HCT116 cells were treated with various concentrations of MHY2245 for 24 h and the cell cycle was analyzed by flow cytometry. As shown in Figure 5A, MHY2245 treatment showed cell cycle arrest at the G2/M phase. After treatment with 1.0 μM MHY2245, 68.85% of cells were in the G2/M phase compared to 34.04% of cells treated with the control (Figure 5B). Subsequently, we confirmed whether MHY2245 regulated the expression of G2/M cell cycle regulators. Cells were treated with increased concentrations of MHY2245 for 24 h, and the levels of G2/M cell cycle-regulating proteins were measured via Western blot analysis. As shown in Figure 5C, the expression of cyclin B1 and its regulatory proteins and cell division cycle protein 2 (Cdc2) and Cdc25c was downregulated in a concentration-dependent manner. Summarizing these experiments, these results clearly indicate that MHY2245 induced cell cycle arrest by regulating the expression of key proteins involved in the regulation of the G2/M phase in HCT116 cells.

### 2.5. Effects of MHY2245 on the Induction of Apoptosis in HCT116 Cells

**Figure 6:**
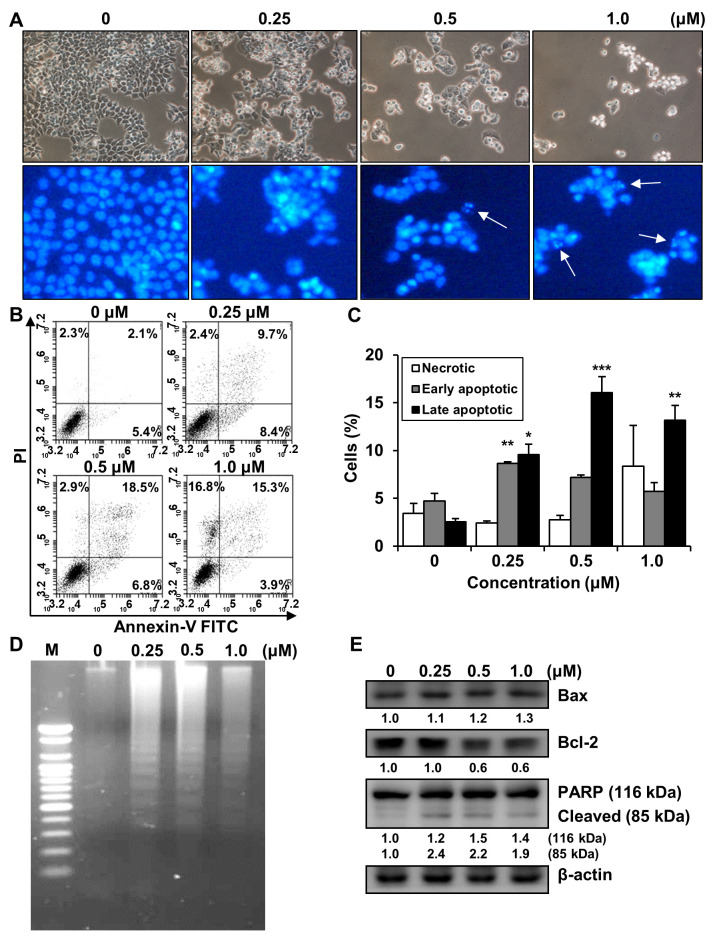
Effect of MHY2245 on the induction of apoptosis in HCT116 cells. (**A**) Morphological changes in MHY2245-treated cells. HCT116 cell nuclei were stained with fluorescent DNA-binding dye (Hoechst 33342). Arrows indicate apoptotic cells (×100). (**B**) Effect of MHY2245 on the death of cells stained with Annexin V-FITC/PI and analyzed via flow cytometry. (**C**) Graph showing the effect of MHY2245 on cell death. Results are presented as mean ± SD, *n* = 3, and are expressed as a percentage of that in vehicle-treated control cells. * *p* < 0.05, ** *p* < 0.01, and *** *p* < 0.001 compared with vehicle-treated control cells. (**D**) Representative results of DNA analyses from three independent experiments are provided. M, marker. (**E**) Effects of MHY2245 on the expression of Bax, Bcl-2, and PARP in HCT116 cells. Western blot analysis of total lysates of cells treated with increasing concentrations of MHY2245. β-actin was used as a loading control. Representative results from three independent experiments are shown.

To address whether the inhibitory effect of MHY2245 is related to apoptotic cell death, morphological changes were assessed. HCT116 cells treated with MHY2245 exhibited distinct morphological changes compared to the untreated controls (Figure 6A, top panel). In addition, a significant reduction in the number of cells was observed in a concentration-dependent manner in HCT116 cells treated with MHY2245. Furthermore, morphological changes in the nuclear structures were analyzed by Hoechst 33342 staining. HCT116 cells showed marked nuclear condensation and shrinkage after MHY2245 treatment (Figure 6A, bottom panel). To verify the apoptotic effects of MHY2245 in HCT116 cells, flow cytometry was performed using Annexin V-fluorescein isothiocyanate (FITC)/propidium iodide (PI) double staining. As shown in Figure 6B,C, the percentage of late apoptotic cells (upper right quadrant, Annexin V-FITC/PI-positive) increased from 2.1% to 18.5% after 24 h exposure to 0.5 μM MHY2245. We investigated the effect of MHY2245 on DNA fragmentation in HCT116 cells, another hallmark of apoptotic cell death. The DNA of cells treated with MHY2245 for 24 h exhibited a characteristic ladder pattern of discontinuous DNA fragments, although untreated control cells showed no signs of fragmentation (Figure 6D). To confirm the apoptotic effects of MHY2245, the expression of apoptosis-related proteins was measured by Western blot analysis. As shown in Figure 6E, the expression of the pro-apoptotic Bcl-2-associated X protein (Bax) was upregulated, and the expression of the anti-apoptotic protein B-cell lymphoma 2 (Bcl-2) was decreased in a concentration-dependent manner. In addition, proteolytic degradation of poly(ADP-ribose) polymerase (PARP), a molecular marker of apoptosis, was induced by treatment with MHY2245. Such findings indicate that MHY2245 induced apoptosis in HCT116 cells.

### 2.6. Effects of MHY2245 on Caspase Activation

**Figure 7:**
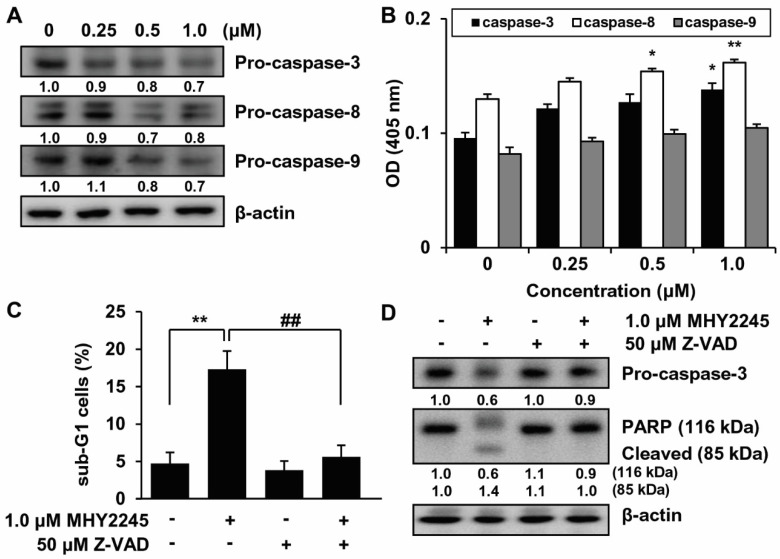
Role of caspases in MHY2245-induced apoptosis. (**A**) The expression of pro-caspases-3, -8, and -9 in MHY2245-treated cells was detected via Western blot analysis. β-actin was used as a loading control. Results from three independent experiments are shown. (**B**) Cell lysates from cells treated with MHY2245 for 24 h were assayed to detect caspase-3, -8, and -9 activities using DEVD-pNA, IETD-pNA, and LEHD-pNA as respective substrates at 37 °C for 1 h. The released fluorescent products were measured. Results are expressed as mean ± SD of three independent experiments. * *p* < 0.05 and ** *p* < 0.01 compared with that in vehicle-treated control cells. (**C**) The presence of cells with sub-G1 DNA content following treatment with MHY2245, indicative of the onset of apoptosis, was detected via flow cytometry. ** *p* < 0.01 compared with that in vehicle-treated control cells. ^##^ *p* < 0.01 compared with that in 1.0 μM MHY2245-treated cells. (**D**) Western blot analysis of PARP and pro-caspase-3 in the total lysates of cells treated with 50 μM Z-VAD-FMK (Z-VAD) and 1.0 μM MHY2245. β-actin was used as a loading control. Representative results from three independent experiments are shown.

We investigated the activation of caspases by MHY2245 in HCT116 cells. HCT116 cells were treated with MHY2245 at various concentrations for 24 h, and changes in the activation of caspases were monitored by Western blot analysis. Caspase is synthesized as an inactive zymogen (pro-caspase) that is activated only after appropriate stimulation. Therefore, the protein levels of pro-caspases were decreased when apoptosis occurs [35]. Protein levels of pro-caspases-3, -8, and -9 were decreased after 24 h of MHY2245 treatment (Figure 7A). Based on these results, the effect of MHY2245 on caspase activity was evaluated using specific substrates. As shown in Figure 7B, HCT116 cells treated with various concentrations of MHY2245 for 24 h showed significantly increased caspase activity in a concentration-dependent manner. To assess the relevance of caspase activation in MHY2245-induced apoptosis, HCT116 cells were cultured in the presence and absence of the pan-caspase inhibitor, Z-VAD-FMK, and analyzed by flow cytometry. As shown in Figure 7C, the percentage of cells that had undergone MHY2245-induced apoptosis in HCT116 cells was decreased by pretreatment with Z-VAD-FMK. This result demonstrates that Z-VAD-FMK prevented the apoptotic effect of MHY2245 in HCT116 cells. Western blotting confirmed these results. Pretreatment with Z-VAD-FMK significantly inhibited the downregulation of MHY2245-induced pro-caspase-3 and PARP cleavage. These results suggest that caspase activation is involved in the induction of MHY2245-induced apoptosis (Figure 7D).

## 3. Discussion

SIRT1 is the most studied enzyme in the SIRT family and is known to regulate cell proliferation, apoptosis, differentiation, migration, and invasion of cancer cells [36,37]. SIRT1 is upregulated in several cancers, and its overexpression can enhance tumor growth and promote cell survival [38,39]. Moreover, SIRT1 overexpression is more frequently observed in advanced-stage colorectal cancers [40,41]. SIRT inhibitors may, therefore, be useful as therapeutic agents, as the overexpression of SIRT has been demonstrated in many human cancer cells [13,42,43,44]. SIRT inhibitors induce DNA strand breaks that lead to DNA damage, cell cycle arrest, and the induction of apoptosis [27,31]. Therefore, in the present study, we evaluated the cytotoxic effect of MHY2245, a sirtinol derivative, on SIRT1/2 inhibition and induction of DNA damage in HCT116 cells. A previous study by Hsu et al. showed that the same HCT116 cells demonstrated an approximately 95% survival rate following treatment with 1.0 μM sirtinol for 48 h [23], and approximately 50% following treatment with 1.0 μM MHY2245 for 48 h (Figure 2A). These results suggest that MHY2245 markedly and more efficiently inhibits the growth of HCT116 cells than sirtinol. In addition, MHY2245 more effectively reduced the cell viability of HCT116 human cancer cell lines than NCM460D, the normal human colon epithelial cell line (Figure 2A,D). Another study found that MHY2245 induces autophagy in human ovarian cancer cells and inhibits energy metabolism through the PKM2/mTOR pathway. Based on the findings of this research group, MHY2245 inhibited tumor growth and reduced tumor size when SKOV3 cells were transplanted into nude mice [45].

DNA damage triggers the activation of DNA damage response elements, such as ATM and ATR [33]. Activation of ATR is usually associated with the initiation of signaling pathways in response to single-strand breaks [46]. We showed that the treatment of HCT116 cells with MHY2245 induced the phosphorylation of ATR protein, but not that of ATM (Figure 4B). Activated ATR phosphorylates downstream proteins, such as H2AX, Chk1, and Chk2, directly or through sequential steps and subsequently affects downstream factors involved in cell cycle progression, cell survival, and/or death [47]. γ-H2AX and p-BRCA1 are involved in DNA repair and the activation of other repair factors, whereas phosphorylated Chk1 and Chk2 activate factors involved in cell cycle arrest and apoptosis [48]. Owing to MHY2245-induced DNA damage in HCT116 cells, H2AX, ATR, Chk1, and Chk2 were found to be highly phosphorylated in this study (Figure 4B).

Cell death is an important pathway for cellular inactivation following DNA damage [49]. The repeated failure of cells to repair DNA lesions eventually leads to DNA double-strand breaks, resulting in cell cycle arrest and cell death [50]. Checkpoints are integrated into DNA repair mechanisms and apoptosis to determine the ultimate reaction of cells to DNA damage [51]. Apoptosis can occur via caspase-dependent and independent mechanisms [52,53]. Mechanistically, sirtinol has been reported to promote apoptosis through changes in the expression of genes involved in apoptotic cell death and activation of caspase cascades [25,26,54,55]. MHY2245-induced activation of caspases in HCT116 cells resulted in significantly decreased cell viability and induction of apoptosis (Figure 7A–D).

In summary, MHY2245 displayed SIRT inhibitory activity, resulting in the induction of DNA damage responses, G2/M phase arrest of the cell cycle, and apoptotic cell death via the caspase pathway in HCT116 cells. As SIRT inhibition and the induction of cancer cell death via the activation of DNA damage responses are common mechanisms of many current chemotherapeutic anticancer drugs, MHY2245 can be further tested in preclinical animal models to assess its anticancer activities against CRC.

## 4. Materials and Methods

### 4.1. Reagents

The simplified code names and structures of MHY2245 are shown in Figure 1. This compound was provided by Professor Hyung Ryong Moon (Pusan National University, Busan, Korea). MHY2245 was synthesized with a yield of 60.8% via the treatment of anthranilamide in MeOH with 1-naphthaldehyde in the presence of a catalytic amount of sulfamic acid at room temperature for 30 min. MHY2245 was dissolved in dimethyl sulfoxide (DMSO) to obtain a 10 mM stock solution and stored at −20 °C until use. Subsequent dilutions were performed in RPMI-1640 (GE Healthcare Life Sciences, Logan, UT, USA). The maximum concentration of DMSO did not exceed 0.1% (*v*/*v*) in the range of treatments that did not affect cell growth. DMSO and 3-(4,5-dimethylthiazol-2-yl)-2,5-diphenyl tetrazolium bromide (MTT) were obtained from Amresco LLC (Solon, OH, USA). Propidium iodide (PI), *N*-acetyl-L-cysteine (NAC), and monoclonal antibody against β-actin were purchased from Sigma-Aldrich (St. Louis, MO, USA). Antibodies specific for p-ataxia telangiectasia mutated kinase (ATM, Ser1981), p-ataxia telangiectasia and Rad3-related kinase (ATR, Ser428), p-checkpoint kinase 1 (Chk1, Ser345), p-Chk2 (Thr68), p-breast cancer type 1 susceptibility protein (BRCA1, Ser1524), and gamma-H2A histone family member X (γ-H2AX, Ser139) were purchased from Cell Signaling Technology (Danvers, MA, USA). Acetylated p53 (Ac-p53, Lys382) was purchased from Abcam (Cambridge, MA, USA). SIRT1, SIRT2, p53, cyclin B1, cell division cycle protein 2 (Cdc2), Cdc25c, B-cell lymphoma 2 (Bcl-2), Bcl-2-associated X protein (Bax), poly(ADP-ribose) polymerase (PARP), caspase-3, -8, -9, and Z-VAD-FMK were obtained from Santa Cruz Biotechnology, Inc. (Dallas, TX, USA).

### 4.2. Docking Simulation of SIRT1/2 and MHY2245

Owing to their automated docking capability, AutoDock Vina, AutoDock 4, and Dock 6 were used for the in silico protein–ligand docking simulation. The 3D structures of SIRT1/2 were used in the crystal structures of human SIRT1 (PDB ID: 4I5I) and SIRT2 (PDB ID: 5YQL). A predefined binding site of SIRT1 [56] and SIRT2 [57] was used as docking pockets. Docking simulations were performed in two sets: (1) between SIRT1 and MHY2245 or splitomicin and (2) between SIRT2 and MHY2245 or AGK2. To prepare compounds for docking simulation, (1) 2D structures were converted into 3D structures, (2) charges were calculated, and (3) hydrogen atoms were added using the ChemSketch program (http://www.acdlabs.com/resources/freeware/chemsketch) (accessed on 5 October 2021). LigandScout 4.1.5 (Inte: Ligand, Vienna, Austria) was used to forecast possible interactions between ligands and SIRT1/2 and to identify pharmacophores.

### 4.3. SIRT1 Deacetylase Activity Assay

The SIRT1 deacetylase activity assay was performed as previously described [58]. SIRT1 activity was evaluated using a SIRT1 Fluorometric Drug Discovery Kit (Enzo Life Sciences, Farmingdale, NY, USA). Briefly, total protein (10 μg) was dissolved in the assay buffer and incubated with the acetylated substrate (Fluor de Lys-SIRT1 substrate, 25 μM) and NAD^+^ (100 μM) at 37 °C for 45 min. The concentration of deacetylated substrates was measured after the addition of the developer, and fluorescence was detected using a fluorescence plate reader (GENios, TECAN Instrument, Salzburg, Austria) with excitation and emission wavelengths of 360 nm and 460 nm, respectively.

### 4.4. Cell Culture and Viability Study

Human HCT116 (WT *TP53*), HT-29 (mutant *TP53*), and DLD-1 (mutant *TP53*) CRC cells were purchased from the American Type Culture Collection (Manassas, VA, USA) and cultured in RPMI-1640 (GE Healthcare Life Sciences) supplemented with 10% fetal bovine serum (GE Healthcare Life Sciences), 100 units/mL penicillin, and 100 μg/mL streptomycin (GE Healthcare Life Sciences) at 37 °C in a humidified atmosphere with 5% CO_2_. The NCM460D cells were provided by Professor Eunok Im (Pusan National University, Busan, Korea). This cell line was incubated in DMEM (GE Healthcare Life Sciences) supplemented with 10% fetal bovine serum (GE Healthcare Life Sciences), 100 units/mL penicillin, and 100 μg/mL streptomycin (GE Healthcare Life Sciences) and cultured with 5% CO_2_ at 37 °C. Cell viability was measured using the MTT assay. Cells were seeded in 24-well culture plates and cultured for 24 h or 48 h before treatment with or without various reagents at the indicated concentrations. Cells were incubated in the dark with MTT (0.5 mg/mL) at 37 °C for 2 h. The formazan granules generated by the live cells were dissolved in DMSO, and the absorbance was measured at 540 nm using a multi-well plate reader (Thermo Fisher Scientific, Waltham, MA, USA).

### 4.5. Cell Cycle Analysis

The effect of MHY2245 on cell cycle regulation was studied as previously described [59]. The DNA content was measured after DNA staining with PI. Cells were treated for 24 h under appropriate conditions, treated with trypsin, washed once with phosphate-buffered saline (PBS), and then treated overnight at −20 °C with 70% ethanol. Fixed cells were pelleted and stained with cold PI solution (50 μg/mL in PBS) in a dark room for 30 min at room temperature. Flow cytometry analyses were performed using an Accuri C6 flow cytometer (BD Biosciences, Franklin Lakes, NJ, USA).

### 4.6. Nuclear Staining with Hoechst 33342

Cells were stained with 4 μg/mL Hoechst 33342 (Life Technologies Corp., Grand Island, NY, USA) at 37 °C for 10 min and examined using a Nikon Eclipse TE 2000-U microscope (Nikon, Tokyo, Japan).

### 4.7. Annexin V-Fluorescein Isothiocyanate (FITC)/PI Double Staining

Annexin V-FITC/PI double staining was tested using flow cytometry as previously described [59]. An Annexin V-FITC apoptosis detection kit (BD Biosciences) was used to quantitatively measure the proportion of cells actively undergoing apoptosis. Cells were harvested, treated with trypsin, washed once with cold PBS, and suspended in binding buffer. Cells were stained with PI and Annexin V-FITC solution at room temperature for 15 min in the dark. The stained cells were analyzed via flow cytometry within 1 h. Flow cytometry analysis was performed using an Accuri C6 flow cytometer (BD Biosciences).

### 4.8. DNA Fragmentation Assay

To find DNA breakage in apoptotic cells, we performed DNA fragmentation assays as previously described [59]. Cells were lysed in ice for 30 min in a buffer containing 5 mM Tris-HCl (pH 7.5), 5 mM ethylenediaminetetraacetic acid (EDTA), and 0.5% Triton X-100. After treating the fragmented DNA with RNase, proteinase K degradation, phenol/chloroform/isoamyl alcohol mixture (25:24:1, *v*/*v*/*v*) extraction and isopropanol precipitation were performed. DNA was isolated from a 1.6% agarose gel, stained with 0.1 μg/mL ethidium bromide, and visualized using an ultraviolet (UV) light source.

### 4.9. Caspase Activity

The activity of caspases was detected as previously described [58]. Cells were incubated with lysis buffer (R&D Systems, Minneapolis, MN, USA) for 10 min on ice. Total protein (100 μg) was incubated with 2X reaction buffer and a substrate of the colorimetric tetrapeptide, including DEVD-pNA for caspase-3, IETD-pNA for caspase-8, and LEHD-pNA for caspase-9. The reaction mixture was incubated at 37 °C for 2 h, and the enzyme-catalyzed release of p-nitroaniline was measured at 405 nm using a multi-well reader (Thermo Fisher Scientific).

### 4.10. Western Blot Analysis

Western blot analysis was performed as previously described [59]. Cells were resuspended in lysis buffer [ 25 mM Tris [pH 7.5], 250 mM NaCl, 5 mM EDTA, 1% nonidet P-40, 100 μg/mL phenylmethylsulfonyl fluoride, and protease inhibitor cocktail]. Equal amounts of protein were denatured by boiling at 100 °C for 5 min in sample buffer (Bio-Rad, Hercules, CA, USA). Total protein was resolved via 8–12% sodium dodecyl sulfate-polyacrylamide gel electrophoresis and transferred to a polyvinylidene difluoride membrane. The membranes were probed with primary antibodies overnight, incubated with horseradish peroxidase-conjugated secondary antibody (Santa Cruz Biotechnology), and visualized using an enhanced chemiluminescence detection system (GE Healthcare Life Sciences, Chicago, IL, UK). The bands from all Western blots were quantified by densitometry using the FluorChem SP Imaging System (Alpha Inotech Corp., San Leandro, CA, USA).

### 4.11. Statistical Analysis

All data analyses were performed using GraphPad Prism version 5.03 (GraphPad Software, San Diego, CA, USA). Results are presented as mean ± standard deviation (SD). Data were analyzed by one-way analysis of variance (ANOVA) for differences between treatments, followed by Tukey’s post hoc test. Statistical significance was set at *p* < 0.05.

## Figures and Tables

**Table 1:** Docking simulation to identify the interactions between the ligand binding domains of SIRT1 and MHY2245.

Compounds	Binding Energy (kcal/mol) ^a^			No. of Van der Waals Bond Interaction Residues ^b^
	AutoDock Vina	AutoDock 4	Dock 6	
Splitomicin (SIRT1 inhibitor)	−9.6	−7.63	−28.46	5
MHY2245	−9.7	−9.7	−33.47	5

**^a^** The Binding energy represents the binding affinity and capacity for the active site of SIRT1 **^b^** The number of Van der Waals bond interaction residues of the enzyme-inhibitor complex were determined using the AutoDock Vina, AutoDock 4, and Dock 6 programs.

**Table 2:** Docking simulation to identify the interactions between the ligand-binding domains of SIRT2 and MHY2245.

Compounds	Binding Energy (kcal/mol) ^a^			No. of Van der Waals Bond Interaction Residues ^b^	No. of Aromatic Interaction Residues ^b^
	AutoDock Vina	AutoDock 4	Dock 6		
AGK2 (SIRT2 inhibitor)	−10.7	−12.7	−36.66	15	1
MHY2245	−10.9	−9.99	−32.00	9	2

**^a^** The Binding energy represents the binding affinity and capacity for the active site of SIRT2. **^b^** The number of Van der Waals bond interaction residues and aromatic interaction residues of the enzyme-inhibitor complex were determined using the AutoDock Vina, AutoDock 4, and Dock 6 programs.

## Data Availability

Data presented in this study are available on request from the corresponding author.
